# Clinical Manifestations of Lyme Borreliosis in Europe: Burden of Lyme Disease Study (BOLD), 2021–2022

**DOI:** 10.3390/pathogens15030327

**Published:** 2026-03-18

**Authors:** Kate Halsby, Alexandra Loew-Baselli, Franc Strle, Anna Moniuszko-Malinowska, Johan Sanmartin Berglund, Viliam Cibik, Dagmar Zakova, Ye Tan, Frederick J. Angulo, Juanita Edwards, Andreas Pilz, Brad D. Gessner, Elizabeth Begier, James H. Stark, on behalf of the BOLD Study Group

**Affiliations:** 1 Pfizer Ltd., Dorking Road, Tadworth, Surrey KT20 7NS, UK; 2 Pfizer Corporation Austria GmbH, Floridsdorfer Hauptstraße 1, 1210 Wien, Austria; alexandra.loew-baselli@pfizer.com (A.L.-B.); andreas.pilz@pfizer.com (A.P.); 3 Department of Infectious Diseases, University Medical Centre Ljubljana, Japljeva 2, 1525 Ljubljana, Slovenia; franc.strle@kclj.si; 4 Department of Infectious Diseases and Neuroinfections, Medical University of Bialystok, ul. Zurawia 14, blok D, 15-540 Bialystok, Poland; annamoniuszko@op.pl; 5 Department of Health, Blekinge Institute of Technology, Valhallavägen 1, 371 79 Karlskrona, Sweden; johan.sanmartin.berglund@bth.se; 6 MUDr. Viliam Cibik, PhD, s.r.o., Vseobecna Ambulancia pre Dospelych, Pruské 293, 018 52 Pruské, Slovakia; viliam.cibik@gmail.com; 7 MUDr. Zakova, s.r.o., Ambulancia Vnutorneho Lekarstva, 911 01 Trenčin, Slovakia; zakova.dagmar@gmail.com; 8 Pfizer Inc., Cambridge, MA 02139, USA; ye.tan@pfizer.com (Y.T.); james.h.stark@pfizer.com (J.H.S.); 9 Pfizer Inc., Collegeville, PA 19426, USA; frederick.j.angulo@pfizer.com (F.J.A.); juanita.edwards@pfizer.com (J.E.); 10 Epidemiology & Vaccinology Consulting Services, Anchorage, AK 99516, USA; brad.gessner@epivac.net; 11 Pfizer Biopharma Group, D04 K7N3 Dublin, Ireland; elizabeth.begier@pfizer.com

**Keywords:** burden of disease, clinical manifestations, disseminated disease, Europe, Lyme borreliosis, Lyme Disease

## Abstract

Lyme borreliosis (LB), the most common European tick-borne disease, can manifest as an erythema migrans (EM) rash or as disseminated LB. The prospective Burden of Lyme Disease (BOLD) study evaluated the frequency of LB clinical manifestations, including signs, symptoms, and treatment patterns in 14 healthcare practices in endemic regions of six European countries: the Czech Republic, Germany, Poland, Slovakia, Slovenia, and Sweden. Between April 2021 and December 2022, patients with suspected LB were evaluated using predefined case definitions that were applied by investigators to identify medically attended LB cases. Enrolled cases were interviewed about their symptoms. Among the 797 LB cases, 615 (77.2%) had EM and 182 (22.8%) had disseminated disease; 154 of the disseminated cases had Lyme arthritis (LA), five had Lyme neuroborreliosis, and three had Lyme carditis. Geographically, the proportion of disseminated disease varied by country, from 1.1% in Slovenia to 78.0% in Slovakia. Overall, 76.3% of all LB cases in Slovakia were LA. Antibiotic use varied by country, although every country prescribed doxycycline. The frequency of LB manifestations varied substantially between countries. EM was the most common manifestation in all countries except Slovakia, where LA was most common. This study underscores the need for improved prevention strategies.

## 1. Introduction

Tick-borne illnesses are a major health concern across Europe, and climate is likely changing the density and range of infection-carrying tick vectors [1]. The most common tick-borne illness in Europe is Lyme borreliosis (LB), which is caused by certain genospecies of *Borrelia burgdorferi* sensu lato [1]. The clinical presentation of LB varies; however, symptoms typically emerge within 30 days of infection. Erythema migrans (EM), a hallmark skin rash observed in most patients with LB, often serves as a key diagnostic factor for localised cases of infection [2,3,4]. Although the majority of LB cases can be effectively treated with an appropriate course of antibiotics [5], those with untreated infection may develop manifestations of disseminated disease [4].

Disseminated LB can impact the musculoskeletal, nervous, and cardiovascular systems, potentially causing serious long-term health effects [2,3,4]. For example, Lyme arthritis (LA) makes up approximately 28% of all LB reported to the Centers for Disease Control and Prevention (CDC) in the United States of America [6,7]. In Europe, that proportion is lower, with a range of estimates varying by country; 0.6% in Slovenia [8], 2.1% in Germany [9], 13.6% in Norway [10], and 17% in Italy [11]. Lyme neuroborreliosis (LNB) can result in painful radiculitis, peripheral facial nerve palsy, and lymphocytic meningitis [12,13], whereas Lyme carditis (LC) usually manifests with heart block [14].

Information on the relative frequency of LB clinical manifestations is limited in Europe due to differences in diagnostic practices and surveillance approaches [8,9,10,15,16,17,18,19,20]. Thus, there is a need for clearer estimation of the frequency of LB manifestations across Europe. To address the challenges posed by differing case definitions and methodologies, the prospective Burden of Lyme Disease (BOLD) epidemiology study was conducted using a standardised surveillance approach and unified case definitions [21]. This methodology permitted comparison of data across endemic regions in 6 European countries: the Czech Republic, Germany, Poland, Slovakia, Slovenia, and Sweden [21]. Data from the BOLD study on the incidence of LB, the diagnostic testing, the patient-reported outcomes, and the cases with Post Treatment Lyme Disease Syndrome will be published elsewhere. Herein, we present the frequency of LB manifestations, including signs and symptoms in BOLD, as well as antibiotic treatment patterns by country. These insights aim to provide a more comprehensive understanding of LB disease burden in six European countries.

## 2. Materials and Methods

### 2.1. Study Design, Setting, and Participants

**Figure 1:**
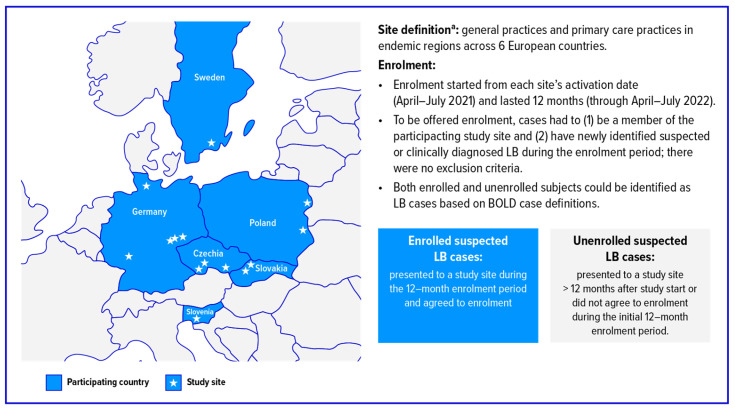
Study Summary. BOLD = Burden of Lyme Disease Study; LB = Lyme borreliosis. ^a^ Although the BOLD methodology paper [21] counted 3 sites separately according to Polish law, 2 were combined for the purpose of the BOLD analysis, as 1 site was the referral site of the other.

Detailed BOLD study methodology has been previously published [21], including site descriptions, surveillance period, and case definitions (Figure 1 and Table S1). In brief, this prospective, active surveillance, epidemiological study was conducted at 14 general practices/primary care practices located in Lyme-endemic areas in the Czech Republic, Germany, Poland, Slovakia, Slovenia, and Sweden. The study sought to identify medically attended LB cases, which include those subjects who sought medical care at their participating clinic and received a clinical diagnosis of EM or a clinical and standard of care laboratory diagnosis of disseminated disease (except multiple EM lesions or disseminated cases with an EM rash, which could be clinically diagnosed) according to the BOLD case definitions (Table S1). Study-site personnel received standardised training on screening and diagnosis of LB using these definitions. Active surveillance and study enrolment began on the activation date for each site, which ranged between April and July 2021, and continued for 12 months. Active surveillance (without enrolment) then continued until the end of 2022. During the initial 12-month period, all patients with suspected LB were offered informed consent before enrolment. Enrolled patients completed a questionnaire on medical history, signs and symptoms, and treatment, and were evaluated at up to five study visits. Visit 1 was at enrolment; visits 2 and 3 occurred 28 days and 10 months, respectively, after visit 1; and visits 4 and 5 were only for LB cases with ongoing symptoms and took place in months 16 to 24 after visit 1. The date of diagnosis was typically the date of visit 1 for newly presenting patients (or could precede the first study visit for cases seen outside the general practitioner’s practice), although it could occur at any time up to visit 2, depending on laboratory diagnostics.

Patients with suspected LB were identified through routine patient tracking or medical records during the active surveillance period. All patients with suspected LB received a final diagnosis of LB (i.e., cases) or “Not LB” based on unified case definitions (Table S1). LB cases were further classified as having disseminated disease if they had LA, LNB, LC, ocular manifestations, acrodermatitis chronica atrophicans, or more than one manifestation. The classification of manifestation was a clinical decision based on the case definitions outlined in the protocol (Table S1), and the final decision was made by primary site investigators. LB diagnoses without established LB clinical manifestations (as defined in Table S1) were also recorded if the subject had a laboratory confirmation. The BOLD study aimed to capture and describe non-traditional presentations of LB and therefore allowed clinical judgement in this more loosely defined group of patients. These cases were given a manifestation classification of “other” and included subjects with, e.g., systemic symptoms or multiple EM.

Local ethics committees for each participating site approved this study, and enrolled participants provided written informed consent [21]. The study complied with legal and regulatory requirements, the International Ethical Guidelines for Biomedical Research Involving Human Participants, the International Council for Harmonisation Good Clinical Practice Guideline, and the Declaration of Helsinki.

### 2.2. Analyses

Analyses were conducted using available data, and subjects with missing data were excluded from the affected analyses. One asymptomatic subject was identified following a routine blood draw for non-study–related purposes and was given an LB diagnosis by site investigators. This subject was excluded from the manifestations, as well as the signs and symptoms analysis. All analyses were descriptive, and no formal statistical hypothesis testing was performed. The analyses were performed using Statistical Analysis System (SAS) software (version 9.4).

## 3. Results

The BOLD study identified 797 medically attended LB cases: 342 in Sweden, 173 in Slovakia, 95 in Slovenia, 94 in the Czech Republic, 63 in Germany, and 30 in Poland (Table S2). Over half (n = 436 [54.7%]) of cases were female, and 331 cases (41.5%) were aged 45–64 years. A total of 315 medically attended LB cases agreed to enrol in the study, with the majority coming from Slovakia (n = 107/315 [34.0%]) and Sweden (n = 104/315 [33.0%]). More females enrolled than males (enrolled: female = 179/315 [56.8%], male = 136/315 [43.2%]; unenrolled: female = 257/482 [53.3%], male = 225/482 [46.7%]), a slightly higher proportion of the enrolled cases fell into the ≥45-year age groups (enrolled 45–64 years = 137/315 [43.5%], ≥65 years = 100/315 [31.7%]; unenrolled 45–64 years = 194/482 [40.2%], ≥65 years = 147/482 [30.5%]), and 33.3% (105/315) of enrolled cases had disseminated disease compared with 16.0% (77/482) of unenrolled cases.

### 3.1. Key Medical History

Medical history was available for each of the 315 enrolled medically attended LB cases, of whom 182 (57.8%) reported a history of tick bites in the years prior to the current LB diagnosis. Of those reporting a history of prior tick bites (lifetime), the median number of tick bite episodes was 10 (interquartile range, 2 to 90). Forty-five subjects reported ≥100 tick bites (44 from Sweden and 1 from Slovenia).

Over a third of enrolled cases had a previous history of LB (n = 112 [35.6%]). Eighty-three cases reported a total of 137 previous EM episodes; of these, 30 were medically confirmed (including 10 laboratory confirmed) and 107 were self-reported. Similarly, 39 enrolled cases reported 46 total previous episodes of disseminated disease; of these, 37 were medically recorded (including 28 laboratory confirmed) and nine were self-reported.

### 3.2. Cases with EM and Disseminated LB

Among the 797 medically attended LB cases, 615 (77.2%) had EM manifestations, 181 (22.7%) had disseminated disease, and one presented asymptomatically (Table S2). More females presented with EM (females = 352/436 [80.7%] and males = 263/360 [73.1%]), whereas more males had disseminated LB (females = 84/436 [19.3%] and males = 97/360 [26.9%]). Most cases of EM and disseminated disease occurred among participants aged ≥45 years (EM: ≥45 years = 437, 0–44 years = 178; disseminated: ≥45 years = 140, 0–44 years = 41). Geographically, over half of the EM cases were reported in Sweden (n = 334), followed by Slovenia (n = 94), the Czech Republic (n = 81), Germany (n = 42), Slovakia (n = 38), and Poland (n = 26). Nearly 75% of the disseminated cases were reported in Slovakia (n = 135), whereas other countries reported far fewer: 21 cases in Germany, 12 in the Czech Republic, eight in Sweden, four in Poland, and one in Slovenia.

### 3.3. Manifestations of LB

**Figure 2:**
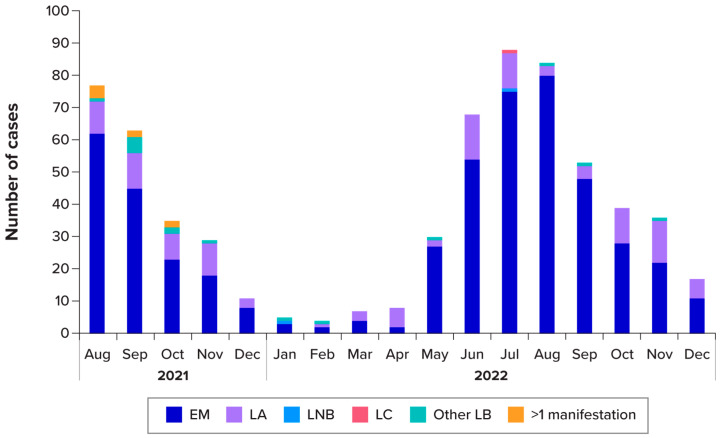
Manifestations by month of diagnosis for 2021 and 2022, counts by subject (unenrolled and enrolled) (n = 796). EM = erythema migrans; LA = Lyme arthritis; LB = Lyme borreliosis; LC = Lyme carditis; and LNB = Lyme neuroborreliosis. Notes: One asymptomatic *Borrelia* infection was not included in the counts for this figure. Seasons were defined as winter (January to March), spring (April to June), summer (July to September), and autumn (October to December). “Other” manifestations are LB cases without an established LB clinical manifestation or with multiple EM.

LB manifestations by subject are summarised in Table 1. Of the 796 LB cases (excluding 1 asymptomatic *Borrelia* infection), 615 (77.3%) had EM, 147 (18.5%) had LA, four (0.5%) had LNB, two (0.3%) had LC, 19 (2.4%) had no established LB clinical manifestations (“other”), and 9 (1.1%) reported more than one manifestation: EM and LA (n = 6); EM and LC (n = 1); EM and LNB (n = 1); and LA and unspecific general symptoms (n = 1). The proportion of subjects presenting with an EM (including those with more than one manifestation) outside Slovakia was 584/623 (93.7%). Both disseminated and EM cases occurred year-round, with most cases diagnosed in the spring and summer months before gradually tapering through the autumn and winter (Figure 2).

**Figure 3:**
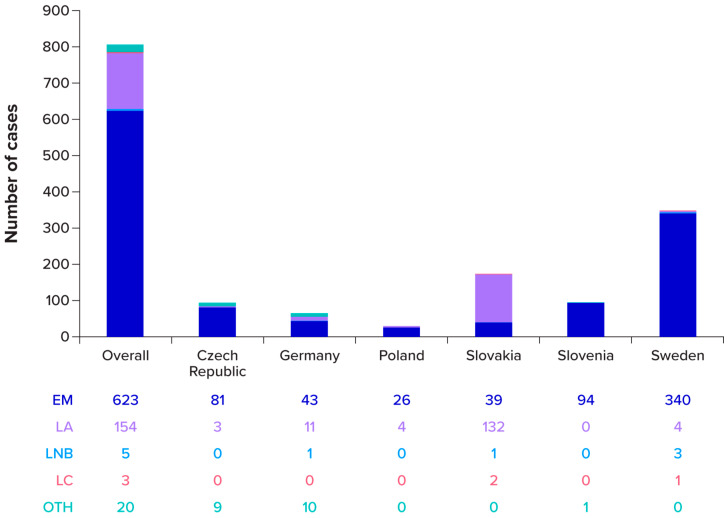
Manifestations by country, counts by manifestation for enrolled and unenrolled subjects (n = 805) EM = erythema migrans; LA = Lyme arthritis; LC = Lyme carditis; LNB = Lyme neuroborreliosis; OTH = other. Notes: One asymptomatic *Borrelia* infection was not included in the counts for this figure. Subjects could have more than one manifestation, which are included individually in this figure. “Other” manifestations are LB cases without an established LB clinical manifestation or with multiple EM.

When individual manifestations are counted (so that subjects with >1 manifestation are counted more than once), there were a total of 805 manifestations among the 796 cases: EM, n = 623 (78.3%); LA, n = 154 (19.3%); LNB, n = 5 (0.6%); LC, n = 3 (0.4%); and no established LB clinical manifestations (“other”), n = 20 (2.5%) (Table 1). These manifestation counts are depicted in Figure 3 by country. For the two most prevalent manifestations, EM and LA, the majority of occurrences were reported in Sweden (n = 340) and in Slovakia (n = 132), respectively.

### 3.4. Signs and Symptoms of LB

**Figure 4:**
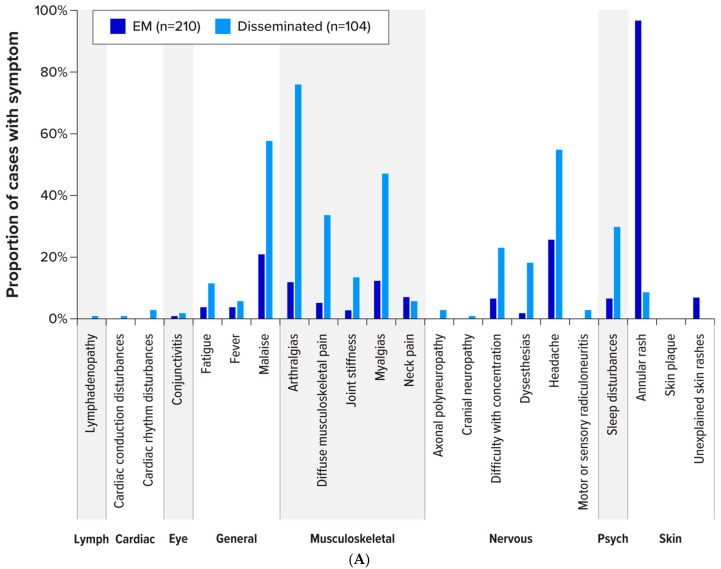

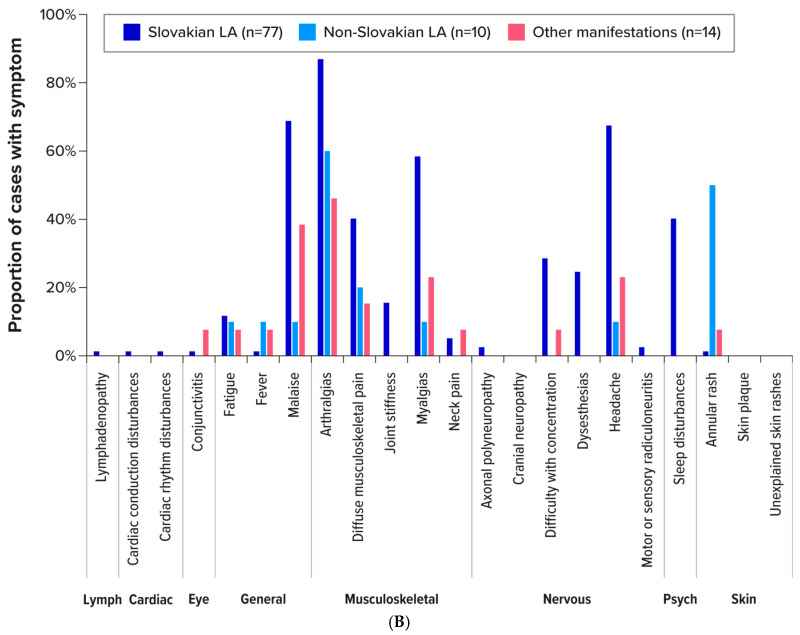
Signs and symptoms among enrolled cases. (**A**) EM and disseminated disease. EM = erythema migrans; Psych = psychiatric disorders. For EM subjects with symptoms potentially indicative of disseminated disease, if no positive laboratory testing was available (not conducted or negative), these cases were clinically diagnosed based on an annular rash and classified as EM. (**B**) Signs and symptoms among cases with LA manifestations, and among LB diagnoses without established LB clinical manifestations (“Other” manifestations), by country. EM = erythema migrans; LA = Lyme arthritis; LB = Lyme borreliosis; Psych = psychiatric disorders. Notes: One asymptomatic *Borrelia* infection was not included in the counts for these figures. “Other” manifestations are LB cases without an established LB clinical manifestation or with multiple EM.

There were 314 enrolled LB cases with information on signs and symptoms (excluding the single asymptomatic infection). Among enrolled cases with a manifestation of EM (n = 210), 203 (96.7%) presented with an annular rash, 15 reported an “unexplained skin rash,” and one reported a “skin plaque.” Other symptoms reported by a large proportion of EM cases included headache (n = 54 [25.7%]) and malaise (n = 44 [21.0%]) (Figure 4A and Table S3). Of enrolled cases with disseminated LB (n = 104), the most commonly reported symptoms were arthralgias (n = 79 [76.0%]), malaise (n = 60 [57.8%]), headache (n = 57 [54.8%]), myalgias (n = 49 [47.1%]), diffuse musculoskeletal pain (n = 35 [33.7%]), sleep disturbances (n = 31 [29.8%]), and difficulty with concentration (n = 24 [23.1%]). Symptoms varied by manifestation (Table S3). Signs and symptoms were recorded for the 12 enrolled cases with no established LB clinical manifestations (“other”); the “other” cases comprised one case with multiple EMs and 11 cases with musculoskeletal and/or general symptoms (Table S3).

LA was the most reported disseminated manifestation among LB cases, and Slovakia had more LA cases than any other country. The signs and symptoms of enrolled LA cases in Slovakia (n = 77) were compared with those of enrolled LA cases outside Slovakia (n = 10) (Figure 4B). Notably, malaise was reported in 68.8% (n = 53) of LA cases in Slovakia (and 10.0% [n = 1] of LA cases outside Slovakia). There were 58 LA cases in Slovakia that presented with nervous system symptoms, including difficulty with concentration (28.6% [n = 22]), dysesthesia (24.7% [n = 19]), and headache (67.5% [n = 52]). The only nervous system symptom reported by LA cases outside Slovakia was headache (10% [n = 1]). One LA case (1.3%) in Slovakia and five LA cases (50%) outside Slovakia reported a rash (all six cases were classified as having more than one manifestation). Cases with LB clinical manifestations that did not fall within pre-specified categories (“other”) reported predominantly generalised and musculoskeletal symptoms with some nervous system symptoms, and one case reported a multiple EM rash (which is considered a disseminated LB manifestation).

The mean time from symptom onset to LB diagnosis was 16.5 days (13.4 days for those with EM and 24.9 days for disseminated disease). Those with “other” manifestations had the longest time from onset to diagnosis (mean, 38.5 days), followed by those with more than one manifestation (mean, 23.8 days) and those with LA (mean, 23.6 days) (Table S4).

### 3.5. Treatment

Among the 315 enrolled LB cases, most were attended by a primary care physician (n = 251 [79.7%]), by a general practitioner (n = 58 [18.4%]), or via telephone consultations with a healthcare provider (n = 40 [12.7%]) when seeking care. Among the other healthcare providers seen were specialists (undefined) (n = 10), rheumatologists (n = 5), dermatologists (n = 4), and neurologists (n = 3) (Table S5). Four cases (1.3%) of 315 enrolled cases were hospitalised (one EM case and three disseminated cases [two with LA and one with LNB]): two cases for one night, and one case each for two nights and three nights.

**Figure 5:**
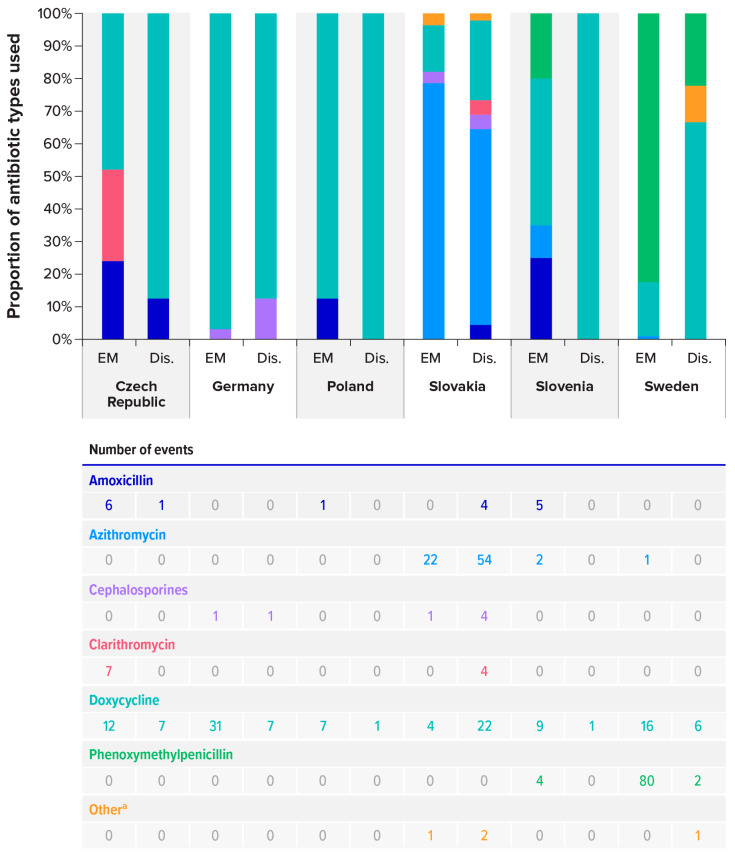
Type of antibiotic treatments prescribed, by country and by subject. Dis. = disseminated; EM = erythema migrans. Note: The table shows event counts. ^a^ Other antibiotics: ciprofloxacin for localised cases in Slovakia, clindamycin and vancomycin for disseminated cases in Slovakia, and flucloxacillin for disseminated cases in Sweden.

Of the 315 enrolled cases, 306 (97.1%) received an antibiotic (three patients refused antibiotics, and six did not receive antibiotic therapy, with no explanation given by the site). The most common types of prescriptions were doxycycline (n = 123 [40.2%]), phenoxymethylpenicillin (n = 86 [28.1%]), and azithromycin (n = 79 [25.8%]). Antibiotic use varied significantly by country, although every country prescribed doxycycline, and it was the most prescribed type of antibiotic in the Czech Republic (57.6%), Germany (95.0%), Poland (88.9%), and Slovenia (47.6%) (Figure 5). In Slovakia, azithromycin was the type of antibiotic most commonly prescribed (64.4%), and in Sweden, phenoxymethylpenicillin was the most common (77.4%). Prescribing patterns for EM cases and disseminated manifestations were similar for all countries, except for Sweden. In Sweden, phenoxymethylpenicillin was most prescribed for EM disease (82.5%), but doxycycline was most prescribed for disseminated disease (66.7%).

Seventeen cases (5.4%) that were prescribed an antibiotic received more than one antibiotic (Slovakia, n = 13; Sweden, n = 2; Czech Republic, n = 1; Poland, n = 1). The most common first-line antibiotic was azithromycin (n = 7), and doxycycline was the most frequently used second-line antibiotic (n = 9). The most frequent combination was azithromycin and doxycycline (n = 6, of whom two received the doxycycline first). The duration of antibiotic use varied by country and by EM or disseminated disease presentation (Table 2). Poland had the longest duration of antibiotic treatment (median, 23.5 days: EM, 22 days; disseminated, 30 days), whereas Slovakia had the shortest (median, 9 days: EM, 9 days; disseminated, 10 days) (Table 2).

## 4. Discussion

The BOLD study is the first prospective epidemiological study for medically attended LB in multiple European countries, evaluating the signs and symptoms and manifestations of LB using standardised case definitions. Overall, over 75% of cases in BOLD had an EM manifestation; when Slovakia is excluded, this increased to 93.7% (584/623). LA was the most common disseminated manifestation, occurring in 19.3% (154/796) of cases (or 3.5% [22/623] excluding Slovakia), whereas LNB (n = 5) and LC (n = 3) case numbers were both very low. The distribution of manifestations in Slovakia was different from that in the other five countries, with LA comprising 76% of total cases. Most patients in the study received at least one course of antibiotic, varying substantially between countries but generally aligned with respective national guidelines.

With the exception of Slovakia, the distribution of manifestations in the BOLD data is similar to that seen in Brestrich et al.’s meta-analysis [18], which combined data from eight studies in France, the Netherlands, Germany, and Slovenia to give the following proportions: EM = 96.9%, LA = 1.3%, LNB = 1.7%, and Other = 0.1%. BOLD (non-Slovakian) data showed a higher proportion of cases without established LB clinical manifestations (“other” manifestations) (3.2%; range, 0% [Poland, Sweden]–15.9% [Germany]), likely due to the study allowing the inclusion of cases with a non-specific symptom presentation that other studies might have excluded. BOLD data also showed a higher proportion of LA cases (3.5%; range, 0% [Slovenia]–17.5% [Germany]) than the 1.3% reported by Brestrich et al. This may have been because final decisions on manifestation classification were made by primary site investigators, and subjects with more generalised systemic symptoms could be inconsistently allocated to either LA or to “other” manifestations. Finally, our LNB data was similar to countries with comprehensive LB surveillance systems, where approximately 2–4% of surveillance-reported LB cases are LNB [20,22,23]. Because the European Centre for Disease Prevention and Control (ECDC) began LNB surveillance in 2018, data on the proportion of LNB cases in Europe have been of good quality.

An unusually high proportion of LA cases were reported in Slovakia, where 76% of cases had LA compared with an average of 3.5% in the other BOLD countries. Although Slovakian LA cases presented with the traditional musculoskeletal symptoms, a large number also presented with general or nervous system symptoms. Additionally, only a few Slovakian LA cases reported an EM rash compared with 50% of the LA cases outside Slovakia. Multiple factors could have played a role in the unusual presentation of LB in Slovakia. There could be differences in the predominant subspecies of *Borrelia burgdorferi* s.l. or a more complex co-infection occurring, or the different presentation may reflect differences in healthcare-seeking behaviour, diagnostic practices, medical adherence, and site-specific clinical focus among different populations. The COVID-19 pandemic may have delayed EM diagnoses, leading to more patients presenting with a disseminated manifestation (including LA). Additionally, the two BOLD study sites in Slovakia were situated in very rural areas, with a local population who may be less likely to attend their primary physician for a skin rash. BOLD is not the first dataset to report a high proportion of LA cases among identified LB cases; a previous Slovakian study (1999–2000) reported 25% of cases were LA [24], and Slovakian public health surveillance data in 2021–2022 reported 8.2% to 17.0% of cases as LA [25,26]. At this time, there is no clear explanation for the higher rate of LA cases reported in Slovakia in this study, and *B. burgdorferi* s.l. isolates from LA cases were not available. It remains possible that there could have been differential classification of disseminated cases by manifestation between countries despite standardised case definitions. With the unusual presentation of LB observed in Slovakia, the laboratory diagnostic practices underpinning the diagnosis are of importance. The BOLD case definition required standard of care laboratory testing for disseminated cases, with Slovakia using a higher proportion of one-tier testing than other countries. The BOLD study also undertook standardised study laboratory testing using a modified two-tier test (MTTT) across all countries, where disseminated cases from the Czech Republic and Slovakia had a lower proportion of positive MTTTs. Laboratory testing in BOLD will be explored more fully in a separate manuscript.

The proportion of subjects presenting with an annular rash in this study was higher than the proportion in the literature [27,28]. This likely reflects a limitation of the data collection methods in BOLD, with no validation performed on these reports to ensure annular rashes were truly ring-shaped. Decisions on signs and symptom reporting were made by local clinical staff, and clinical judgement was accepted. Clinicians may have preferentially considered annular rashes as diagnostic of EM, while homogeneous rashes may have been less frequently recognised or classified as such. The large number of tick bites reported in some subjects’ medical histories was also unexpected; the maximum reported was 999. Data entry was capped at the value 999, and other high values were entered (e.g., 800, 600), so the large values are plausible. All but one of the values above 100 were reported by the Swedish cases, indicating that the Swedish study site was situated in an area with frequent tick exposure.

Almost all patients in the study received at least one course of an antibiotic. The types of antibiotics prescribed generally aligned with respective national guidelines (Appendix B), with minor variations that may be due to physician decisions based on patients’ medical history and needs. The variation in the type of antibiotic used by each country shows that there are multiple antibiotics still effective against LB, and only 17/306 (6%) cases required >1 antibiotic. Most of the study sites had mean and median antibiotic treatment times that were within their country recommendations and were based on prescribing guidelines (e.g., azithromycin has a shorter prescription length than most other antibiotics recommended for LB). For EM cases, the mean and median treatment times were slightly longer than their respective country guidelines for the Czech Republic, Germany, Poland, and Sweden. However, some antibiotic prescriptions require doses more than once per day, and if the first dose was given late in the day, a 14-day course could have been spread across 15 days. Some patients received a very high number of days of antibiotics to treat their LB, which can come with adverse effects, such as antibiotic resistance, microbiome disruption, and *Clostridioides difficile* infection [29,30]. The three longest courses of antibiotics were 48, 53, and 93 days. All three were disseminated cases, two qualified for a classification of PTLDS, and the third reported symptoms of disease lasting 16 months. The persistent symptoms and PTLDS cases will be described in a separate manuscript. An effective Lyme vaccine could have an important impact in reducing this antibiotic exposure.

This study has limitations. The results reported here for sites selected among high LB incidence regions in Europe may not be generalisable to other locations. Generalised conclusions were also limited by the uneven contribution of cases across countries, limiting cross-national comparability. Clinicians at the BOLD sites may be more experienced at identifying and diagnosing LB, potentially identifying more unusual cases with higher accuracy than clinicians would elsewhere. Similarly, antibiotic prescribing practices for the treatment of LB cases in the BOLD sites may not be representative of nationwide prescribing practices in each country. The clinical manifestations of LB may vary geographically based on the local prevalence of different *Borrelia* genospecies. Diagnostic and treatment practices vary across countries, which may also influence how and when manifestations are reported. Laboratory confirmation for the case definition was based on standard-of-care testing, which varied between sites and countries. There was potential for misdiagnosis, especially of EM cases where a laboratory confirmation was not required. In particular, the high proportion of annular rash presentations compared with prior literature suggests some misclassification. The presentation of LB cases to healthcare clinics in 2021 may have been affected, and healthcare-seeking behaviour may have been delayed as a result of the coronavirus disease (COVID-19) pandemic in 2020. Not all LB cases were willing to enrol in the study, or presented during a period when enrolment was no longer open, so signs and symptom descriptions and medical history are available only for the enrolled subset, which was slightly older, more likely to be female, and more likely to have disseminated disease. This may have affected the signs and symptoms reported in the dataset (with potentially more systemic and fewer localised cases in older age groups) and may have led to more use of antibiotics for disseminated disease. All analyses were descriptive, which limits the strength of inferences. There was an uneven contribution of cases by country, limiting the possibility of cross-country comparisons.

## 5. Conclusions

These findings show that the frequency of LB manifestations varies substantially between countries, with the distribution differing markedly in Slovakia. This study also improves understanding of treatment patterns across Europe and highlights the need for prevention measures, such as vaccination. To continue supporting clinical trials and public health needs, additional studies are needed to further characterise LB clinical manifestations across Europe and to understand drivers for between-country variation.

## Figures and Tables

**Table 1:** Manifestations overall and by country (enrolled and unenrolled).

		By Subject						By Manifestation				
	Overall, n	EM, n (%)	LA, n (%)	LNB, n (%)	LC, n (%)	“Other,” ^a^ n (%)	>1 Manifestation, ^b^ n (%)	EM, n (%)	LA, n (%)	LNB, n (%)	LC, n (%)	“Other,” ^a^ n (%)
All	796	615 (77.3)	147 (18.5)	4 (0.5)	2 (0.3)	19 (2.4)	9 (1.1)	623 (78.3)	154 (19.3)	5 (0.6)	3 (0.4)	20 (2.5)
Czech Republic	93	81 (87.1)	3 (3.2)	0	0	9 (9.7)	0	81 (87.1)	3 (3.2)	0	0	9 (9.7)
Germany	63	42 (66.7)	9 (14.3)	1 (1.6)	0	9 (14.3)	2 (3.2)	43 (68.3)	11 (17.5)	1 (1.6)	0	10 (15.9)
Poland	30	26 (86.7)	4 (13.3)	0	0	0	0	26 (86.7)	4 (13.3)	0	0	0
Slovakia	173	38 (22.0)	131 (75.7)	1 (0.6)	2 (1.2)	0	1 (0.6)	39 (22.5)	132 (76.3)	1 (0.6)	2 (1.2)	0
Slovenia	95	94 (98.9)	0	0	0	1 (1.1) ^c^	0	94 (98.9)	0	0	0	1 (1.1)
Sweden	342	334 (97.7)	0	2 (0.6)	0	0	6 (1.8)	340 (99.4)	4 (1.2)	3 (0.9)	1 (0.3)	0

EM = erythema migrans; LA = Lyme arthritis; LC = Lyme carditis; and LNB = Lyme neuroborreliosis. Note: One asymptomatic *Borrelia* infection was not included in the counts for this table. The denominator for percentage calculations is the total number of cases in each country. ^a^ “Other” manifestations are LB cases without an established LB clinical manifestation or with multiple EM. ^b^ Some subjects had multiple manifestations: EM and LA (n = 6); EM and LC (n = 1); EM and LNB (n = 1); and LA and other (n = 1). ^c^ Multiple EM.

**Table 2:** Duration of antibiotic use by subject (in days).

	n	Median	Mean	Min	Max
Czech Republic	30	18	17	7	27
EM	24	16	17	7	27
Disseminated	6	23	20	11	27
Germany	40	20	18	3	34
EM	32	20	19	9	34
Disseminated	8	20	17	3	31
Poland	8	24	25	20	32
EM	7	22	25	20	32
Disseminated	1	30	30	30	30
Slovakia	100	9	16	2	93
EM	26	9	12	2	31
Disseminated	74	10	18	3	93
Slovenia	21	15	15	5	24
EM	20	15	15	5	24
Disseminated	1	14	14	14	14
Sweden	103	11	12	6	24
EM	96	11	11	6	21
Disseminated	7	22	19	11	24
**Total**	**302**	**11**	**15**	**2**	**93**

EM = erythema migrans; max = maximum; min = minimum. Note: Event counts are shown. Some antibiotic prescriptions require doses more than once per day, and if the first dose was given late in the day, a 14-day course could have been spread across 15 days. The number of treatment cycles or the time between cycles was not captured. Four subjects were not included in the duration calculation; all terminated their study involvement after visits 1 or 2, with antibiotic courses marked as “ongoing” and no further follow-up possible.

## Data Availability

Upon request, and subject to review, Pfizer will provide the data that support the findings of this study. Subject to certain criteria, conditions, and exceptions, Pfizer may also provide access to the related individual de-identified participant data. See https://www.pfizer.com/science/clinical-trials/trial-data-and-results (accessed on 9 March 2026) for more information.

## Supplementary Materials

The following supporting information can be downloaded at: https://www.mdpi.com/article/10.3390/pathogens15030327/s1. Table S1. Lyme borreliosis case definitions; Table S2. Demographics of EM and disseminated cases, by subject; Table S3. Symptoms of enrolled LB cases by manifestation; Table S4. Manifestations by time from onset to clinical diagnosis for enrolled subjects (in days); Table S5. Number and type of medical visits. Ref. [31] is cited in supplementary material.

## Appendix A

Investigators of the Burden of Lyme Disease (BOLD) Study Group

▪ Dr. Lenka Dybova; Doktor Brno, S.R.O.; Brno, Czech Republic;
▪ Dr. Miroslav Pavlasek; ADMED, S.R.O.; Ceske Budejovice, Czech Republic;
▪ Dr. Daniela Verdanova; Ordinace praktickeho lekare pro deti a dorost; Jindrichuv Hradec, Czech Republic;
▪ Dr. Joachim Weimer; Private Practice of Dr. Joachim Weimer; Reinfeld, Germany;
▪ Dr. Christine Kosch; Private Practice of Dr. Christine Kosch; Pirna, Germany;
▪ Dr. Kai Mehltretter; Velocity Clinical Research; Leipzig, Germany;
▪ Dr. Hans-Detlev Stahl; AmBeNet GmbH; Leipzig, Germany;
▪ Dr. Barbara Nieradko-Iwanicka; Lubelskie Centrum Diagnostyczne; Swidnik, Poland and Medical University of Lublin, Lublin, Poland;
▪ Dr. Dorota Bielska; Akademicka Praktyka Medycyny Rodzinnej; Bialystok, Poland;
▪ The BOLD study group also included Drs Berglund, Moniusko, Strle, Cibik, and Zakova, who are included in the co-authorship list.

## Appendix B

**Table A1:** Detailed summary of antibiotic treatment guidelines by country.

Clinical Form	Czech Republic [32]	Germany [33,34,35]	Poland [36]	Slovakia [37]	Slovenia [38]	Sweden [39,40]
EM	Preferred: ▪ AMOX ^a,b^ ([adult: 500–1000 mg × 3; child: 50 mg/kg × 1], oral, 10–14 d) ▪ CLA ([adult: 500 mg × 2; child: 15 mg/kg × 1], oral, 10–14 d) ▪ **CXMA** ([adult: 500 mg × 2; child: 30 mg/kg × 1], oral, 10–14 d) ▪ DOX ^a,d,h^ ([adult: 100 mg × 2 or 200 mg × 1; child: 4 mg/kg × 1], oral, 10–14 d) ▪ PV ^b^ ([adult: 1–1.5 MIU × 3; child: 80–100 TIU/kg × 1], oral, 10–14 d) Other: ▪ AZM ^d^ (adult: 500 mg × 2, oral, 1 d; then 500 mg × 1, oral, 4 d [child: 20 mg/kg, oral, 1 d; then 10 mg/kg, oral, 4 d])	Recommended: ▪ AMOX ^c^ ([adult: 500–1000 mg × 3; child: 50 mg/kg × 1], oral, 14 d) ▪ DOX ([adult ^f^: 100 mg × 2 or 200 mg × 1; child ^g^: 4 mg/kg × 1, max 200 mg × 1], oral, 10–14 d) Other: ▪ AZM ([adult: 250 mg × 2; child: 5–10 mg/kg × 1], oral, 5–10 d) ▪ CXMA ([adult: 500 mg × 2; child: 30–50 mg/kg × 1], oral, 14 d)	Adults: Preferred: ▪ AMOX (500 mg × 3, oral, 14–21 d) ▪ CXMA^c^ (500 mg × 2, oral, 14–21 d) ▪ DOX (100 mg × 2 daily or 200 mg daily, oral or IV, 7–21 d) Other: ▪ AZM ^c^ (500 mg × 1, oral, 5–10 d) Children: ▪ AMOX (50 mg/kg in 3 divided doses, max 500 mg per dose, oral, 14 d) ▪ AZM ^c^ (10 mg/kg × 1, max 500 mg per dose, oral, 5–10 d) ▪ CXMA ^c^ (30 mg/kg in 2 divided doses, max 500 mg per dose, oral, 14 d) ▪ DOX ^h^ (4.4 mg/kg in 2 divided doses, max 200 mg daily, oral or IV, 10 d)	Recommended: ▪ AMOX ([adult: 500 mg × 3; child: 30–50 mg/kg × 1], oral, 10–21 d) ▪ CXMA ([adult: 500 mg × 2; child: 30–40 mg/kg × 1], oral, 10–21 d) ▪ DOX ^g^ ([adult: 100 mg × 2 or 200 mg × 1; child: 4 mg/kg × 1], oral, 10–21 d) ▪ PV ([adult: 1–1.5 MIU × 3; child: 0.1 MIU/kg × 1], oral, 10–21 d) Other: ▪ AZM ([adult: 500 mg × 1; child 10 mg/kg × 1], day 1 double dose, oral, 5–10 d) ▪ CLA ([adult: 500 mg × 2; child: 7.5 mg/kg × 1], oral, 10–21 d)	Recommended: ▪ AMOX (adult: 500–1000 mg × 3, oral, 14 d) ▪ CXM (adult: 500 mg × 2, oral, 14 d) ▪ DOX ^b,d^ (adult: 100 mg × 2, oral, 10–14 d) ▪ PV (adult: 1–1.5 MIU × 3, oral, 14 d) Other: ▪ AZM ^c,e^ (adult: 500 mg × 2, oral, 1 d; then 500 mg × 1, oral, 4 d) ▪ CRO ^f^ (adult: 2 g × 1, IV, 14 d) ▪ PG ^c^ (adult: 5 MIU × 4, IV, 14 d) Children: ▪ AMOX (50 mg/kg × 3, max 500 mg per 8 h, oral, 10–30 d) ▪ AZM (20 mg/kg × 2 for 1 d, then 10 mg/kg 2–5 d, max 1000 mg/kg at 1 d and 500 mg/kg at 2–5 d, oral, 5 d) ▪ CLA (15 mg/kg × 2, max 500 mg per 12 h, oral, 10–30 d) ▪ CXMA (30 mg/kg × 2, max 500 mg per 12 h, oral, 10–30 d) ▪ DOX ^h^ (4 mg/kg × 2, max 100 mg per 12 h, oral, 10–30 d) ▪ PV (10,000 IU/kg × 3, max 1 million IU per 8 h, oral, 10–30 d)	Recommended: ▪ AZM ^d,e^ (adult: 500 mg × 1, oral, 1 d; then 250 mg × 1, oral, 4 d [child: 10 mg/kg × 1, oral, 1 d; then 5 mg/kg × 1, oral, 4 d]) ▪ DOX ^c,d^ ([adult: 100 mg × 2 or 200 mg × 1; child: 4 mg/kg × 1, max 200 mg × 1], oral, 10 d) ▪ PV ([adult: 1 g × 3 or 2 1 g × 3 ^c^ 4 ^f^; child: 25 mg/kg × 3, max 1 g × 3], oral, 10 d)
Multiple EM	• AZM ^d^ (adult: 500 mg × 2, oral, 1 d; then 500 mg × 1, oral, 4 d [child: 20 mg/kg, oral, 1 d; then 10 mg/kg, oral, 4 d]) • CLA ([adult: 500 mg × 2; child: 15 mg/kg × 1], oral, 10–14 d) • CXMA ([adult: 500 mg × 2; child: 30 mg/kg × 1], oral, 10–14 d) • PV ^b^ ([adult: 1–1.5 MIU × 3; child: 80–100 TIU/kg × 1], oral, 10–14 d)	• AMOX ^i,j^ ([adult: 500–1000 mg × 3; child: 50 mg/kg × 1], route N.S., 14–21 d) • AZM ^i,j^ ([adult: 250 mg × 2; child: 5–10 mg/kg × 1], route N.S., 5–10 d) • CXMA ^i,j^ ([adult: 500 mg × 2; child: 30–50 mg/kg × 1], route N.S., 14–21 d) • DOX ^i,j^ ([adult ^f^: 100 mg × 2 or 200 mg × 1; child ^g^: 4 mg/kg × 1, max 200 mg × 1], route N.S., 14–21 d)	• N.S.	• CRO ([adult: 2 g × 1; child 50–75 mg/kg × 1], IV or IM, 14–21 d) • CTX ([adult: 2 g × 3; child 150–200 mg/kg × 1], IV or IM, 14–21 d) • DOX ^g^ ([adult: 200–400 mg × 1; child: 4–8 mg/kg × 1], oral, 14–21 d) • PG ([adult: 18–24 MIU × 1; child 0.25–0.5 MIU/kg × 1], IV, 14–21 d)	• CRO (adult: 2 g × 1, IV, 14 d) • DOX ^b,h^ (adult: 100 mg × 2, oral, 14 d)	• AMOX (child [<8 years old]: 15 mg/kg × 3, oral, 10–14 d) • AZM ^c^ (child: 10 mg/kg × 1, oral, 1 d; then 5 mg/kg × 1, oral, 4 d) • CRO ^c,k,f^ (adult: 2 g × 1, IV, 10 d) • DOX ^e,h,k^ (adult: 100 mg × 2 or 200 mg × 1, oral, 10 d [child: 4 mg/kg × 1, max 200 mg × 1, oral, 10–14 d])
Borrelial lymphocytoma	• AZM ^d^ (adult: 500 mg × 2, oral, 1 d; then 500 mg × 1, oral, 4 d [child: 20 mg/kg, oral, 1 d; then 10 mg/kg, oral, 4 d]) • CLA ([adult: 500 mg × 2; child: 15 mg/kg × 1], oral, 10–14 d) • CXMA ([adult: 500 mg × 2; child: 30 mg/kg × 1], oral, 10–14 d) • PV ^b^ ([adult: 1–1.5 MIU × 3; child: 80–100 TIU/kg × 1], oral, 10–14 d)	• Same as multiple EM	Adults: ▪ AMOX (500 mg × 3, oral, 14–21 d) ▪ DOX (100 mg × 2 or 200 mg × 1, oral or IV, 14–21 d) ▪ CXMA ^c^ (500 mg × 2, oral, 14–21 d) Children: ▪ AMOX (50 mg/kg in 3 divided doses, max 500 mg per dose, oral, 14–21 d) ▪ DOX ^h^ (4.4 mg/kg in 2 divided doses, max 200 mg daily, oral or IV, 14–21 d) ▪ CXMA ^c^ (30 mg/kg in 2 divided doses, max 500 mg per dose, oral, 14–21 d)	• Same as EM	• Same as EM	• AMOX (child: 15 mg/kg × 3, oral, 14 d) • AZM ^c^ (child: 10 mg/kg × 1, oral, 1 d; then 5 mg/kg × 1, oral, 4 d) • DOX ^d,e^ ([adult: 100 mg × 2 or 200 mg × 1; child: 4 mg/kg × 1, max 200 mg × 1], oral, 14 d) • PV ([adult: 1 g × 3; child: 25 mg/kg × 3, max 1 g × 3], oral, 14 d)
Acrodermatitis chronica atrophicans	• CRO ([adult: 2 g × 1; child: 50–75 mg/kg × 1], IV, 14–28 days) • CTX ([adult: 2 g × 3; child: 150–200 mg/kg × 1], IV, 14–28 d)	Without neurological symptoms: ▪ AMOX ([adult: 500–1000 mg × 3; child: 50 mg/kg × 1], route N.S., 30 d) ▪ DOX ([adult ^f^: 100 mg × 2 or 200 mg × 1; child ^g^: 4 mg/kg × 1; max 200 mg × 1], route N.S., 30 d) With neurological symptoms: ▪ CRO ^k^ ([adult: 2 g × 1; child: 50 mg/kg × 1], route N.S., 14–21 d) ▪ CTX ^k^ ([adult: 2 g × 3; child: 100–150 mg/kg × 1], route N.S., 14–21 d) ▪ PG ^k^ ([adult: 5 MIU × 4; child: 200–500,000 IU/kg × 1], route N.S., 14–21 d)	Adults: ▪ AMOX (500 mg × 3, oral, 21–28 d) ▪ CXMA ^c^ (500 mg × 2, oral, 21–28 d) ▪ DOX (100 mg × 2 or 200 mg × 1, oral or IV, 21–28 d) Children: ▪ N.S.	• AMOX ([adult: 500 mg × 3; child: 30–50 mg/kg × 1], oral, 21 d) • CRO ([adult: 2 g × 1; child: 50–75 mg/kg × 1], IV or IM, 21 d) • CTX ([adult: 2 g × 3; child: 150–200 mg/kg × 1], IV or IM, 21 d) • CXMA ([adult: 500 mg × 2; child: 30 mg/kg × 1], oral, 21 d) • DOX ^g^ ([adult: 200 mg × 1 or 100 mg × 2; child: 4–8 mg/kg × 1], oral, 21 d)	• CRO (adult: 2 g × 1, IV, 21 d) • DOX ^b,d^ (adult: 100 mg × 2, oral, 28 d)	• DOX ^d^ (adult: 100 mg × 2 or 200 mg × 1, oral, 21 days) • PV (adult: 2 g × 3, oral, 21 d)
Neuroborreliosis	Recommended: ▪ CRO ([adult: 2 g × 1; child: 50–75 mg/kg × 1], IV, 14–28 d) Other: ▪ CTX ([adult: 2 g × 3; child: 150–200 mg/kg × 1], IV, 14–28 d) ▪ DOX ^a,d,h^ ([adult: 100 mg × 2 or 200 mg × 1; child: 4 mg/kg × 1], oral, 10–14 d) ▪ PG ([adult: 4–5 MIU every 4–6 hr; child: 200–400 TIU/kg × 1], IV, 14–28 days)	Early: ▪ CRO ([adult: 2 g × 1; child: 50 mg/kg × 1], IV, 14 d) ▪ CTX ([adult: 2 g × 3; child: 100 mg/kg × 1], IV, 14 d) ▪ DOX ([adult ^f^: 100 mg × 2–3 or 200–300 mg × 1; child: 4 mg/kg × 1, max 200 mg/kg × 1], oral, 14 d) ▪ PG ([adult: 5 MIU × 4; child: 200–500,000 IU/kg × 1], IV, 14 d) Late: ▪ Same as for early neuroborreliosis, except duration for all antibiotics ranges from 14 to 21 d	Adults: Preferred: ▪ CRO (2 g × 1, IV, 14–21 d) ▪ CTX (2 g × 3, IV, 14–21 d) ▪ DOX (100 mg × 2 or 200 mg × 1, oral or IV, 14–21 d) Other: ▪ PG (18– mln j/d in 6 divided doses, IV, 14–21 d) Children: ▪ CTX ^h^ (150–200 mg/kg in 3–4 divided doses [max 6 g × 1, IV], 14–21 d)	Early: ▪ Same as multiple EM Late: ▪ CRO ([adult: 2 g × 1; child: 50–75 mg/kg × 1], IV or IM, 21 d) ▪ CTX ([adult: 2 g × 3; child: 150–200 mg/kg × 1], IV or IM, 21 d) ▪ DOX ^g^ ([adult: 200–400 mg × 1; child: 4–8 mg/kg × 1], oral, 21 d) ▪ PG ([adult: 4–5 MIU × 1; child: 200,000–400,000 IU/kg × 1], route N.S., 21 d)	Early: ▪ CRO ^l^ (adult: 2 g × 1, IV, 14 d) ▪ DOX ^b,d^ (adult: 100 mg × 2, oral, 14 d) Late: ▪ CRO ^l^ (adult: 2 g × 1, IV, 14–28 d)	• CRO (adult: 2 g × 1, IV, 14 d [child: 50–100 mg/kg × 1, IV, 10 d]) • DOX ^c,d^ (adult: 200 mg × 1, oral, 14 d; or 200 mg × 2, oral, 10 d [child: 4 mg/kg × 1, max 200 mg × 1, oral, 10 d])
Lyme arthritis	• AMOX ^a^ ([adult: 500–1000 mg × 3; child: 50 mg/kg × 1], oral, 10–14 d) • CRO ([adult: 2 g × 1; child: 50–75 mg/kg × 1], IV, 14–28 d) • CTX ([adult: 2 g × 3; child: 150–200 mg/kg × 1], IV, 14–28 d) • CXMA ([adult: 500 mg × 2; child: 30 mg/kg × 1], oral, 10–14 d) • DOX ^a,d,h^ ([adult: 100 mg × 2 or 200 mg × 1; child: 4 mg/kg × 1], oral, 10–14 d)	• N.S.	Adults: First episode: ▪ AMOX (500 mg × 3, oral, 28 d) ▪ CXMA ^c^ (500 mg × 2, oral, 28 d) ▪ DOX (100 mg × 2 or 200 mg × 1, oral or IV, 28 d) For recurrence: ▪ CRO (2 g × 1, IV, 14–28 d) Children: First episode: ▪ AMOX (50 mg/kg in 3 divided doses, max 500 mg per dose, oral, 28 d) ▪ DOX ^h^ (4.4 mg/kg in 2 divided doses, max 200 mg daily, oral or IV, 28 d) ▪ CXMA ^c^ (30 mg/kg in 2 divided doses, max 500 mg per dose, oral, 28 d) For recurrence: ▪ CRO (50–75 mg/kg × 1, 2 g max per dose, IV, 14–28 d)	Late • CRO ([adult: 2 g × 1; child: 50–75 mg/kg × 1], IV or IM, 28 d) • CTX ([adult: 2 g × 3; child: 150–200 mg/kg × 1], IV or IM, 28 d) • DOX ^g^ ([adult: 200 mg × 1 or 100 mg × 2; child: 4–8 mg/kg × 1], oral, 28 d)	• CRO (adult: 2 g × 1, IV, 14 d) • DOX ^b,d^ (adult: 100 mg × 2, oral, 28 d)	• AMOX (child: 15 mg/kg × 3, oral, 21 d) • CRO (adult: 2 g × 1, IV, 14 d) • DOX ^d,h^ (adult: 100 mg × 2, oral, 14 d; or 200 mg × 1, oral, 28 d [child: 4 mg/kg × 1, max 200 mg × 1, oral, 21–28 d])
Lyme carditis	Recommended: ▪ CRO ([adult: 2 g × 1; child: 50–75 mg/kg × 1], IV, 14–28 d) ▪ CTX ([adult: 2 g × 3; child: 150–200 mg/kg × 1], IV, 14–28 d) Other: ▪ AMOX ^a^ ([adult: 500–1000 mg × 3; child: 50 mg/kg × 1], oral, 10–14 d) ▪ CXMA ([adult: 500 mg × 2; child: 30 mg/kg × 1], oral, 10–14 d) ▪ DOX ^d,h^ ([adult: 100 mg × 2 or 200 mg × 1; child: 4 mg/kg × 1], oral, 10–14 d)	• N.S.	Adults: Recommended: ▪ DOX ^h^ (100 mg × 2 or 200 mg × 1, oral or IV, 14–21 d) ▪ AMOX (500 mg × 3, oral, 14–21 d) ▪ CXMA ^c^ (500 mg × 2, oral, 14–21 d) ▪ CRO (2 g × 1, IV, 14–21 d) Other: ▪ CTX (2 g × 3, IV, 14–21 d) ▪ PG (18– mln j/d in 6 divided doses, IV, 14–21 d) Children: ▪ AMOX (50 mg/kg in 3 divided doses, max 500 mg per dose, oral, 14–21 d) ▪ CRO ^h^ (50–75 mg/kg, max 2 g per dose, IV, 14–21 d) ▪ CXMA ^c^ (30 mg/kg in 2 divided doses, max 500 mg per dose, oral, 14–21 d) ▪ DOX ^h^ (4.4 mg/kg in 2 divided doses (max 200 mg daily), IV or oral, 14–28 d)	• Same as multiple EM	Mild: ▪ DOX ^b,d^ (adult:100 mg × 2, oral, 14 d) ▪ AMOX (adult: 500–1000 × 3, oral, 14 d) Severe: ▪ CRO ^m^ (adult: 2 g × 1, IV, 14 d) Children: ▪ Same as EM	• CRO ([adult: 2 g × 1; child [<5 years old]: 50 mg/kg × 1], IV, 14 d) • DOX ^d^ ([adult: 100 mg × 2 or 200 mg × 1; child [>5 years old]: 4 mg/kg × 1, max 200 mg × 1], oral, 14 days)
Ocular manifestations	• 14–21 d of treatment, dosage N.S.	• N.S.	• CRO ^h^ (50–75 mg/kg ounces per day, max 2 g per dose, IV, duration N.S.) • PG (200–400 TIU/kg in 6 divided doses, max 18–24 MIU per day, IV, duration N.S.) • DOX ^h^ (4.4 mg/kg in 2 divided doses, max 200 mg daily, oral or IV, duration N.S.)	• Same as multiple EM	• CRO (adult: for most forms of ocular involvement)	• CRO (adult: 2 g × 1; child [<5 years old]: 50 mg/kg × 1], IV, 21 d) • DOX ([adult: 200 mg × 1; child [<5 years old]: 4 mg/kg × 1, max 200 mg × 1], oral, 21 days)

AMOX = amoxicillin; AZM = azithromycin; CLA = clarithromycin; CRO = ceftriaxone; CTX = cefotaxime; CXM = cefuroxime; CXMA = cefuroxime axetil; d = day(s); DOX = doxycycline; EM = erythema migrans; IM = intramuscular; IV = intravenous; MIU = million international units; N.S. = not specified; PG = penicillin G (benzylpenicillin); PV = penicillin V (phenoxymethylpenicillin); and TIU = thousand international units. Note: This summary table is based on published guidance. For more details, including context or evidence grading (when applicable), please refer to the cited references. ^a^ For “Skin” LB. ^b^ Not suitable during breastfeeding. ^c^ In case of penicillin allergy. ^d^ Restrictions apply during pregnancy. ^e^ During breastfeeding. ^f^ During pregnancy. ^g^ For those greater than 8 years of age (Poland: without inflammatory changes in the cerebrospinal fluid). ^h^ Not recommended in children under 8 years of age. ^i^ Includes EM and fever or flu-like symptoms (for Germany). ^j^ In case of multiple EM and Borrelia lymphocytoma, treatment duration is 21 days. ^k^ Oral administration can be continued up to 30 days. ^l^ PG (20 MIU × 1) or CTX (2 g every 8 h) are equally as effective. ^m^ In case of improvement, treatment can be completed orally.

**Table A2:** High-level overview of antibiotic treatment guidelines by country.

	Czech Republic [32]		Germany [33,34,35]		Poland [36]		Slovakia [37]		Slovenia [38]		Sweden [39,40]	
Antibiotic	EM	Dis.	EM	Dis.	EM	EM	EM	Dis.	EM	Dis.	EM	Dis.
AMOX												
AZM												
CLA												
CRO												
CTX												
CXM												
CXMA												
DOX												
PG												
PV												

AMOX = amoxicillin; AZM = azithromycin; CLA = clarithromycin; CRO = ceftriaxone; CTX = cefotaxime; CXM = cefuroxime; CXMA = cefuroxime axetil; DOX = doxycycline; Dis. = disseminated disease; EM = erythema migrans; PG = penicillin G (benzylpenicillin); and PV = penicillin V (phenoxymethylpenicillin). Note: Antibiotics included in country-specific LB treatment guidelines are indicated by blue-coloured cells. EM disease columns cover recommendations for EM, whereas disseminated disease columns cover all other manifestations, including multiple EM. This high-level overview does not specify recommendations by age group, disease severity, or otherwise. For more details, please see Table A1 and the provided references.

### Summary

Treatment guidelines are summarised in Table A1 and Table A2. Across the 6 countries represented in the BOLD study (the Czech Republic, Germany, Poland, Slovakia, Slovenia, and Sweden), there is broad agreement regarding first-line therapy for early Lyme borreliosis (LB), particularly for erythema migrans (EM). For EM, amoxicillin and doxycycline are widely recommended as the first-line agents for adults in all countries, whereas antibiotics like azithromycin serve as alternatives across countries. For paediatric patients, doxycycline is generally reserved for children aged 8 years and older. Most guidelines recommend a treatment duration of 10 to 21 days for early LB, with some variation depending on the drug selected and the clinical context. Some guidelines specify different durations for EM versus disseminated disease or recommend repeating oral therapy or switching to intravenous therapy if symptoms persist.

## Appendix C

**Table A3:** Ethics committee approval information.

Country	Ethics Committee	Initial Approval Date	Initial Approval Code
Czech Republic	Eticka komise IKEM a FTN Fakultni Thomayerova nemocnice Videnska 800 Praha 4—Krc, 140 59	16 February 2021	12/21+4217/21
Germany	Ethik-Kommission bei der Landesärztekammer Hessen Hanauer Landstrasse 152 Frankfurt am Main, 60314	12 March 2021	2021-2414-zvBO
Germany	Ethikkommission bei der Ärztekammer Schleswig-Holstein Bismarckallee 8–12 Bad Segeberg, 23795	12 March 2021	034/21 m
Germany	Ethikkommission bei der Sächsischen Landesärztekammer Schützenhöhe 16 Dresden, SACHSEN 01099	12 March 2021	EK-BR-22/21-1
Poland	Komisja Bioetyczna przy Lubelskiej Izbie Lekarskiej w Lublinie ul. Chmielna 4 Lublin, 20-079	1 February 2021	140/2021/KB/VIII
Poland	Komisja Bioetyczna przy Uniwersytecie Medycznym w Bialymstoku ul. Jana Kilinskiego 1 Bialystok, 15-089	25 March 2021	APK.002.169.2021
Poland	Komisja Bioetyczna przy Okregowej Izbie Lekarskiej z siedziba w Bialymstoku ul. Swietojanska 7 Bialystok, 15-082	21 April 2021	12/2021/VIII
Slovakia	Eticka Komisia Trencianskeho samospravneho Karja K dolnej stanici 7282/20A Trencin, 911 01	10 February 2021	No code provided—the approval references Pfizer’s protocol number: C4601002
Slovenia	Komisija Republike Slovenije za medicinsko etiko Stefanova 5 Ljubljana, 1000	31 May 2021	0120-53/2021-4
Sweden	Etikprövningsmyndigheten Box 2110 Uppsala, 750 02	5 May 2021	2021-00604
