# Supplementary material for: Clinical Manifestations of Lyme Borreliosis in Europe: Burden of Lyme Disease Study (BOLD), 2021–2022
Source: Pathogens. 2026 Mar 18;15(3):327. doi: 10.3390/pathogens15030327 (PMC13029277; doi:10.3390/pathogens15030327)
Supplement: Supplementary file 1 [file pathogens-15-00327-s001.zip › pathogens-4141950-supplementary.pdf]

Supplementary Material

Table S1. Lyme Borreliosis Case Definitions

Presentation	Sign/Symptom	Laboratory Diagnostic(s)
Erythema migrans	Characteristic red or bluish-red patch, with or without central clearing	▪ Positive IgG/IgM on serum Bbsl antibody testing OR<br>▪ Positive PCR of Bbsl result from biopsy OR<br>▪ Positive culture of Bbsl from biopsy
Borrelial lymphocytoma	Painless bluish-red nodule or plaque, usually on ear lobe, ear helix, nipple, or scrotum	
Acrodermatitis chronica atrophicans	Long-standing red or bluish-red lesions, usually on the extensor surfaces of extremities. Initially doughy swelling. Possible skin induration and fibroid nodules over bony prominences	
Lyme neuroborreliosis	Meningo-radiculoneuritis (Bannwarth syndrome), facial palsy, meningitis, encephalomyelitis, OR cerebral vasculitis	Intrathecal Bbsl IgM and/or IgG antibodies OR<br>Positive intrathecal Borrelia antibody index (CSF versus serum) reflecting intrathecal Bbsl antibody production OR<br>Positive PCR of Bbsl result from CSF OR<br>Positive culture of Bbsl from CSF
Lyme carditis	Acute onset of high degree atrioventricular conduction disturbances, rhythm disturbances, myocarditis, OR pancarditis	Positive IgG/IgM on serum Bbsl antibody testing
Lyme arthritis	Marked swelling in 1 or few large joints, most often the knee	Positive IgG/IgM on serum Bbsl antibody testing OR<br>Positive PCR of Bbsl result from synovial fluid or tissue OR<br>Positive Culture of Bbsl from synovial fluid or tissue
LB ocular manifestations	Conjunctivitis, uveitis, papillitis, episcleritis, OR keratitis	Positive IgG/IgM on serum Bbsl antibody testing OR<br>Positive PCR of Bbsl result from ocular fluid OR<br>Positive culture of Bbsl from ocular fluid

Bbsl = *Borrelia burgdorferi* sensu lato; CSF = cerebrospinal fluid; IgG = immunoglobulin G; IgM = immunoglobulin M; LB = Lyme borreliosis; PCR = polymerase chain reaction.

Note: Case definitions developed from the European Union Concerted Action on Lyme Borreliosis (EUCALB) definitions [15,31].

Table S2. Demographics of EM and Disseminated Cases, by Subject

	Total (n=797)	Enrolled cases (n=315)			Unenrolled cases (n=482)		Unenrolled and enrolled (n=797)		
		EM (n=210)	Disseminated (n=104)	Asymptomatic (n=1)	EM (n=405)	Disseminated (n=77)	EM (n=615)	Disseminated (n=181)	Asymptomatic (n=1)
Country									
Czech Republic	94	24	7	1	57	5	81	12	1
Germany	63	33	9	0	9	12	42	21	0
Poland	30	8	1	0	18	3	26	4	0
Slovakia	173	28	79	0	10	56	38	135	0
Slovenia	95	20	1	0	74	0	94	1	0
Sweden	342	97	7	0	237	1	334	8	0
Sex									
Female	436	133	46	0	219	38	352	84	0
Male	361	77	58	1	186	39	263	97	1
Age group, years									
0-19	51	10	1	0	39	1	49	2	0
20-44	168	40	27	0	89	12	129	39	0
45-64	331	86	50	1	157	37	243	87	1
65+	247	74	26	0	120	27	194	53	0

EM = erythema migrans.

Table S3. Symptoms of Enrolled LB Cases by Manifestation

		By subject n (%)							Total (n=314)		Symptom duration (in days)	
Category	Symptom	EM (n=210)	LA (n=80)	LNB (n=2)	LC (n=1)	Other (n=12)	>1 Manifestation (n = 9)[a]	Disseminated (n=104)	n	n with duration[b]	Mean (SD)	Median (min, max)
Blood/lymph	Lymphadenopathy	1 (0.5)	1 (1.3)	0	0	0	0	1 (1.0)	2	2	22.5 (20.5)	22.5 (8, 37)
Cardiac	Cardiac conduction disorders	0	1 (1.3)	0	0	0	0	1 (1.0)	1	1	140.0 (NE)	140.0 (140, 140)
	Cardiac rhythm disturbances	1 (0.5)	1 (1.3)	0	1 (100)	0	1 (11.1)	3 (2.9)	4	2	402.0 (531.7)	402.0 (26, 778)
Eye disorders	Conjunctivitis	2 (1.0)	1 (1.3)	0	0	1 (8.3)	0	2 (1.9)	4	3	46.7 (11.0)	46.0 (36, 58)
General disorders	Fatigue	8 (3.8)	8 (10.0)	0	0	1 (8.3)	3 (33.3)	12 (11.5)	20	7	356.6 (310.0)	327.0 (17, 781)
	Fever	8 (3.8)	1 (1.3)	1 (50.0)	0	1 (8.3)	3 (33.3)	6 (5.8)	14	13	18.1 (25.1)	5.0 (2, 71)
	Malaise	44 (21.0)	53 (66.3)	1 (50.0)	0	4 (33.3)	2 (22.2)	60 (57.7)	104	81	130.6 (188.8)	47.0 (1, 781)
MSK and connective tissue disorders	Arthralgias	25 (11.9)	70 (87.5)	0	0	5 (41.7)	4 (44.4)	79 (76.0)	104	63	193.6 (216.6)	66.0 (11, 899)
	Diffuse MSK pain	11 (5.2)	31 (38.8)	1 (50.0)	0	1 (8.3)	2 (22.2)	35 (33.7)	46	32	113.4 (152.1)	49.0 (1, 614)
	Joint stiffness	6 (2.9)	12 (15.0)	0	0	0	2 (22.2)	14 (13.5)	20	14	197.1 (290.7)	47.5 (7, 743)

Category	Symptom	By subject n (%)							Total (n=314)		Symptom duration (in days)	
		EM (n=210)	LA (n=80)	LNB (n=2)	LC (n=1)	Other (n=12)	>1 Manifestation (n = 9)[a]	Disseminated (n=104)	n	n with duration[b]	Mean (SD)	Median (min, max)
	Myalgias	26 (12.4)	44 (55.0)	1 (50.0)	0	2 (16.7)	2 (22.2)	49 (47.1)	75	57	125.9 (173.1)	46.0 (1, 725)
	Neck pain	15 (7.1)	4 (5.0)	0	0	1 (8.3)	1 (11.1)	6 (5.8)	21	16	100.5 (195.4)	33.5 (1, 739)
Nervous system disorders	Axonal polyneuropathy	0	2 (2.5)	1 (50.0)	0	0	0	3 (2.9)	3	2	387.5 (480.1)	387.5 (48, 727)
	Cranial neuropathy	0	0	0	0	0	1 (11.1)	1 (1.0)	1	0	NE	NE
	Difficulty with concentration	14 (6.7)	22 (27.5)	1 (50.0)	0	1 (8.3)	0	24 (23.1)	38	31	140.9 (200.8)	50.0 (4, 764)
	Dysesthesias	4 (1.9)	18 (22.5)	0	0	0	1 (11.1)	19 (18.3)	23	20	128.9 (215.9)	49.0 (7, 731)
	Headache	54 (25.7)	52 (65.0)	1 (50.0)	0	3 (25.0)	1 (11.1)	57 (54.8)	111	83	77.7 (128.5)	37.0 (1, 731)
	Motor or sensory radiculoneuritis	1* (0.5)	1 (1.3)	1 (50.0)	0	0	1 (11.1)	3 (2.9)	4	4	264.3 (277.9)	212.0 (19, 614)
Psychiatric disorders	Sleep disturbances	14 (6.7)	31 (38.8)	0	0	0	0	31 (29.8)	45	38	141.8 (163.2)	57.5 (24, 731)
Skin and subcutaneous tissue disorders	Annular rash	203 (96.7)	0	0	0	1 (8.3)	8 (88.9)	9 (8.7)	212	191	30.9 (20.1)	29.0 (1, 142)
	Skin plaque	1 (0.5)	0	0	0	0	0	0	1	1	30.0 (NE)	30.0 (30, 30)
	Unexplained skin rashes	15 (7.1)	0	0	0	0	0	0	15	13	23.9 (12.5)	22.0 (3, 44)

EM = erythema migrans; LA = Lyme arthritis; LC = Lyme carditis; LNB = Lyme neuroborreliosis; max = maximum; min = minimum; MSK = musculoskeletal system; NE = not estimable; SD = standard deviation.

Note: One asymptomatic *Borrelia* infection was not included in the counts for this table.

[a] EM/LA [n=6], EM/LC [n=1], EM/LNB [n=1], LA/Other [n=1].[b] Symptom duration calculated as (Date of diagnosis – Date of onset of first reported symptom of LB) + 1. A duration could not be calculated for subjects who had symptoms that were ongoing at their time of exiting the study.

*Subject had negative standard of care laboratory testing; therefore, only a clinical diagnosis of EM was possible.

Table S4. Manifestations by Time From Onset to Clinical Diagnosis[a] for Enrolled Subjects (in Days)

Clinical manifestations	Total, n	n in calculation[b]	Days		
			Mean (SD)	Median (Q1, Q3)	Min, max
All subjects	314	287	16.5 (26.5)	8.0 (3.0, 18.0)	1, 233
Erythema migrans	210	209	13.4 (18.0)	7.0 (3.0, 15.0)	1, 121
All disseminated	104	78	24.9 (40.4)	14.0 (4.0, 30.0)	1, 233
Lyme neuroborreliosis	2	2	11.0 (7.1)	11.0 (6.0, 16.0)	6, 16
Lyme arthritis	80	56	23.6 (36.8)	14.0 (4.5, 22.5)	1, 176
Lyme carditis	1	1	1.0 (0.0)	1.0 (1.0, 1.0)	1, 1
Other	12	10	38.5 (70.1)	16.5 (3.0, 39.0)	1, 233
>1 manifestation[c]	9	9	23.8 (22.9)	12.0 (6.0, 43.0)	1, 62

EM = erythema migrans; LA = Lyme arthritis; LC = Lyme carditis; LNB = Lyme neuroborreliosis
max = maximum; min = minimum; Q1 = quartile 1; Q3 = quartile 3; SD = standard deviation.

Note: One asymptomatic *Borrelia* infection was not included in the counts for this table.

[a] Time from symptom onset to clinical diagnosis is calculated as (date of diagnosis − earliest start date of symptom) +1.

[b] For subjects with known dates of onset and diagnosis.

[c] EM/LA [n=6], EM/LC [n=1], EM/LNB [n=1], LA/Other [n=1].

Table S5. Number and Type of Medical Visits

Type of medical visit	Number of subjects with at least 1 medical visit, n (%)	Frequency of medical visits[a]
Emergency room	4 (1.3)	4
Primary care physician	251 (79.7)	277
Alternative medicine or therapy	1 (0.3)	1
Cardiologist	0	0
Dermatologist	4 (1.3)	4
Family medicine	2 (0.6)	2
Internal medicine	2 (0.6)	2
Neurologist	3 (1.0)	3
Rheumatologist	5 (1.6)	6
General practitioner	58 (18.4)	63
Specialist	10 (3.2)	10
Internist	0	0
Hospital clinic	2 (0.6)	2
Urgent care	0	0
Telephone consultation	40 (12.7)	47
Other	7 (2.2)	10

[a] The total number of visits to a type of practitioner attended throughout the study.
